# Macrophage Tropism in Pathogenic HIV-1 and SIV Infections

**DOI:** 10.3390/v12101077

**Published:** 2020-09-25

**Authors:** Matthew Moeser, Joshua R. Nielsen, Sarah B. Joseph

**Affiliations:** 1 Lineberger Comprehensive Cancer Center, University of North Carolina at Chapel Hill, Chapel Hill, NC 27599, USA; moeser@ad.unc.edu; 2 Department of Microbiology and Immunology, University of North Carolina at Chapel Hill, Chapel Hill, NC 27599, USA; jrniels@med.unc.edu

**Keywords:** SIV, HIV-1, myeloid cells, macrophage, CD4, entry, NHP, tropism

## Abstract

Most myeloid lineage cells express the receptor and coreceptors that make them susceptible to infection by primate lentiviruses (SIVs and HIVs). However, macrophages are the only myeloid lineage cell commonly infected by SIVs and/or HIVs. The frequency of infected macrophages varies greatly across specific host and virus combinations as well as disease states, with infection rates being greatest in pathogenic SIV infections of non-natural hosts (i.e., Asian nonhuman primates (Asian NHPs)) and late in untreated HIV-1 infection. In contrast, macrophages from natural SIV hosts (i.e., African NHPs) are largely resistant to infection due to entry and/or post-entry restriction mechanisms. These highly variable rates of macrophage infection may stem from differences in the host immune environment, entry and post-entry restriction mechanisms, the ability of a virus to adapt to efficiently infect macrophages, and the pleiotropic effects of macrophage-tropism including the ability to infect cells lacking CD4 and increased neutralization sensitivity. Questions remain about the relationship between rates of macrophage infection and viral pathogenesis, with some evidence suggesting that elevated levels of macrophage infection may contribute to greater pathogenesis in non-natural SIV hosts. Alternatively, extensive infection of macrophages may only emerge in the context of high viral loads and immunodeficiency, making it a symptom of highly pathogenic infections, not a primary driver of pathogenesis.

## 1. Introduction

Human immunodeficiency viruses (HIV-1 and HIV-2) and simian immunodeficiency viruses (various SIVs) are primate lentiviruses capable of generating chronic disease in humans and in nonhuman primates (NHPs). SIVs have been replicating in African nonhuman primates for at least 32,000 years [1] and are now highly diverse, with over 40 African NHP species infected with a species-specific SIV [2]. In contrast, HIV-1 and HIV-2 emerged much more recently as separate zoonotic transmissions of SIVs from African primates to humans [3]. Around 1920, the transmission of SIVcpz from chimpanzees to humans generated pandemic HIV-1 [4], which has resulted in tens of millions of deaths. The less pathogenic HIV-2 jumped to humans in the mid-1900s by multiple transmissions of SIV from sooty mangabeys (SIVsm) [5].

The development of antiretroviral therapy (ART) and increased knowledge of HIV biology have saved millions of lives. Much of this progress was made possible by the use of NHP models of HIV infection. SIV infections of African NHPs (their natural hosts) typically generate no disease or disease that is much less pathogenic than HIV-1 infections in humans [6], making these natural hosts poor models of HIV pathogenesis. Fortuitously, after being housed in US primate centers with African NHPs, Asian macaques developed immunodeficiency similar to that of HIV-infected people. Eventually, these Asian macaques, which are non-natural hosts of SIVs, were found to have acquired SIV, and the observed disease pathogenesis was similar to that of HIV-infected people, although accelerated [7]. Many NHP models of HIV infection have been developed in which species of Asian macaques are infected with various SIVs.

All primate lentiviruses replicate extensively in CD4+ T-cells, and certain variants are also able to efficiently infect macrophages [8,9,10,11,12,13,14,15,16,17,18]. Macrophage infection is of particular interest given that some macrophages may be able to live for decades [19], making it possible for infected cells to persist for long periods of time and serve as a barrier to curing HIV-infected humans. Furthermore, severe neurocognitive impairment in HIV-infected people may involve viral replication and adaptation in macrophages [12]. Finally, infection of macrophages has been proposed to be a major contributor to overall disease pathogenesis [20]. Addressing these hypotheses has been challenging because macrophages reside in tissues that are difficult to sample in humans. Thus, NHP models are necessary to careful characterize primate lentiviral infection of macrophages.

This review will explore SIV and HIV-1 infection of myeloid lineage cells, particularly macrophages, which are the only myeloid lineage cell type infected at appreciable rates [21,22,23]. Rates of macrophage infection can reach high levels in the brain during pathogenic infections [20,24] but are typically low. We will focus on humans infected with HIV-1 and SIV-infected Asian macaques (non-natural hosts of SIV), the two systems in which infection of macrophage has been most thoroughly characterized. Additionally, we will examine mechanistic differences in how SIVs and HIVs adapt to infect macrophages. We hypothesize that an improved understanding of macrophage-tropism in SIVs and HIVs may reveal important information about the immune environments in which macrophage-tropic variants evolve and their potential contributions to viral pathogenesis.

## 2. Primate Lentiviruses Primarily Replicate in CD4+ T-Cells

Entry is the first step in infection and is one of the primary determinants of cellular tropism. Primate lentivirus entry (reviewed by [25]) is facilitated by the viral Env protein, which is expressed as a trimer on the virion surface. Entry begins with the binding of the viral Env protein to CD4 on the host cell. After binding to the CD4 surface receptor, Env undergoes conformational changes that expose the V3 loop and form the bridging sheet. The V3 loop and bridging sheet then mediate binding to the coreceptor, and the Env trimer undergoes additional conformational changes that facilitate the fusion of the viral and host cell membranes, allowing the viral capsid to enter the cell.

Given that entry involves the sequential binding of CD4 and a coreceptor, primate lentiviral infection is typically restricted to cells expressing CD4 and a coreceptor (CCR5 or CXCR4, or alternative coreceptors in NHPs) at densities that support viral entry. Studies have repeatedly shown that antibodies or drugs altering viral interactions with CD4 inhibit viral entry and replication in vivo [26,27,28,29] and in vitro [26,30], indicating that the CD4 receptor is typically needed for HIV-1 and SIV replication. In humans and in NHPs, CD4 is primarily expressed on CD4+ T-cells [31,32] and myeloid lineage cells (e.g., monocytes, macrophages, and dendritic cells (DCs) [31,33,34]). Table 1 summarizes receptor and coreceptor expression on these potential target cells in humans and in Asian NHPs.

### 2.1. SIVs and HIV-1 Primarily Replicate in CD4+ T-Cells

Of their potential target cells, CD4+ T-cells are the primary cells in which HIV-1 and SIVs replicate. The preferential infection of CD4+ T-cells is clearly supported by studies of infected humans and NHPs in which the frequency of CD4+ T-cells containing viral DNA, RNA, or protein is high relative to the low frequency found in monocytes in the blood [21,22] or myeloid cells in tissues [48,49,50]. This is also supported by in vitro studies showing that most HIV-1 and SIV variants enter and replicate more efficiently in CD4+ T-cells relative to macrophages [14,48,51,52]. The extremely rapid depletion of CD4+ T-cells from gut-associated lymphatic tissue very early in infection [53,54,55,56,57,58] also reflects the targeting of CD4+ T-cells. However, it is important to note that some of this depletion is likely due to bystander killing, not viral infection (reviewed by [59]). Together, these data indicate that CD4+ T-cells are the primary target cells in SIV and HIV-1 infections.

### 2.2. Most Myloid Lineage Cells Are Largely Resistant to HIV-1 and SIV Infection

Myeloid lineage cells are granulocytes, monocytes, macrophages, and dendritic cells (DCs) [60]. These cells have phagocytic and inflammatory responses to pathogens as well as protective roles in maintaining tissue homeostasis, repairing damage, and influencing development. The majority of myeloid cells are produced by common myeloid progenitors in the bone marrow; however, a subset of tissue-resident macrophages (e.g., microglia in the brain and Kupffer cells in the liver) are produced by erythromyeloid progenitors that colonize tissues during fetal development [61,62].

Monocytes and DCs have been proposed to be potential targets for SIV and HIV replication, but there is no evidence that they are frequently infected [21,22,23]. While DCs are not directly infected, virions have been observed on the surface or in endosomes of DCs, particularly follicular dendritic cells (fDCs) [63,64], and DCs can transmit virions to CD4+ T-cells [65]. Similarly, monocytes express CD4, albeit at very low levels [31], yet are largely resistant to HIV-1 infection in vitro [66] and infected monocytes are rarely found in the blood of HIV-infected people on [22] or off ART [21]. The resistance of DCs and monocytes to infection is likely derived from both pre-entry blocks (e.g., very low levels of CD4 or CCR5) and post-entry blocks (discussed below).

### 2.3. Restriction Factors Limiting Viral Infection in Myeloid Lineage Cells

Primate lentiviral infection of myeloid lineage cells is thought to be limited by the host protein SAMHD1 (sterile alpha motif and histidine–aspartate domain-containing protein 1), which impairs reverse transcription of the viral RNA genome by reducing nucleotide pools within resting CD4+ T-cells and myeloid cells [67,68]. HIV-2 and some SIV variants, including those derived from SIVsm, express the Vpx protein, which targets SAMHD1 for proteasome-mediated degradation, thus increasing viral replication in myeloid cells and in resting CD4+ T-cells [68,69]. HIV-1 and some other SIVs lack the *vpx* gene but express the accessory protein Vpr, which may also inhibit SAMHD1 [70].

Studies examining the role that SAMHD1 plays in infection of myeloid cells suggest that SAMHD1 restriction alone does not explain the extremely low levels of myeloid cell infection in vivo. One experiment comparing macaques infected with either an SIV expressing Vpx or with a *vpx* deletion mutant found that the Vpx expressing SIV infected macrophages at higher rates, suggesting that Vpx does enhance infection of myeloid lineage cells in vivo [71]. However, Vpx also increased the overall pathogenesis of infections, making it difficult to ascertain whether elevated rates of macrophage infection were mediated by Vpx degradation of SAMHD1 or overall disease pathogenesis. In contrast, another study comparing infections initiated with SIVmac239 and SIVmac239Δvpx observed that expression of Vpx had little effect on the amount of DNA in myeloid lineage cells and found that much of the viral DNA detected in macrophages was due to the phagocytosis of infected T-cells [48]. The low levels of infection observed in myeloid cells, even in the presence of Vpx, could be explained by an inability of Vpx to overcome SAMHD1 completely [72], other post-entry restriction factors, or by CD4 and/or coreceptor densities that are too low to facilitate efficient entry.

## 3. HIV-1 Infection of Macrophage

### 3.1. HIV-1 Primarily Infects Macrophages When CD4+ T-Cells Are Limited

The frequency of macrophage infection is highly variable in HIV-infected people but is consistently higher in pathogenic infections late in the disease. This is most clearly illustrated by studies examining HIV-1 populations in the immune-privileged CNS at end-stage disease. Immunostaining of brain tissue from such individuals has identified large numbers of productively infected microglia [73,74,75,76] and CNS macrophages [73,77]. HIV-1 infection of macrophages in the CNS is also supported by genetic analyses of virus populations in the cerebrospinal fluid (CSF) and blood. Early in infection, HIV-1 populations in the blood and CSF are typically closely related [78], but as the disease progresses, genetically distinct viral populations (i.e., compartmentalized populations) can emerge in the CSF [9,11,12]. Studies have also found that unlike populations in the blood, compartmentalized populations in the CSF are occasionally well adapted to infecting macrophages/microglia [8,9,10,11,12,13,79,80,81,82] (in this review, we will not deal with the important question of whether CNS macrophages and microglia are both targets for HIV-1 infection and, if so, are they equally infected). Such compartmentalized, macrophage-tropic populations emerge due to sustained viral replication and adaptation in macrophages/microglia in the CNS, a phenotype that may be favored in the CNS where CD4+ T-cells are rare and late in disease when CD4+ T cell counts are low. HIV-1 has also been observed to infect alveolar macrophages in the lungs [83], urethral macrophages in penile tissue [84], and liver macrophages [85].

### 3.2. Macrophage-Tropic HIV-1 Has an Enhanced Ability to Infect Cells Expressing a Low Surface Density of CD4

Multiple studies have shown that the vast majority of HIV-1 variants throughout the body, and virtually all HIV-1 variants in the blood, are poorly adapted to infecting macrophages (i.e., are T-cell-tropic) and require high CD4 densities similar to the levels on CD4+ T-cells for efficient entry ([86,87]; reviewed in [88]). However, a small subset of HIV-1 variants is able to efficiently enter macrophages (i.e., are macrophage-tropic) and has an enhanced ability to infect cells expressing a low surface density of CD4 [9,11,12,79,80,81,82]. This phenotype facilitates the infection of macrophages that express a low density of CD4 on their surface [31,89].

Macrophage-tropic HIV-1 can occasionally be isolated from humans, and these variants are able to efficiently infect cells expressing low CD4 densities, but in vitro analyses indicate that they do not have the ability to efficiently infect cells lacking CD4 [89]. This is difficult to reconcile with observations that HIV-1 occasionally infects kidney epithelial cells [90,91] and astrocytes [92,93,94,95], both of which lack CD4. The most likely explanation is that HIV-1 infects these cells via a highly inefficient mechanism that allows HIV-1 to enter cells without first binding CD4. The efficiency of CD4-independent entry can be improved by passaging HIV-1 in tissue culture [96,97,98,99]. The resulting tissue-culture-adapted variants have been observed to have an enhanced ability to infect cells lacking CD4 and an open Env conformation that greatly increases their neutralization sensitivity, which likely explains why they are not observed in vivo [100,101]. Together, these results indicate that it is possible for HIV-1 to evolve the ability to efficiently infect cells lacking CD4, but that natural selection does not favor this phenotype in humans.

## 4. Infection of Macrophages in Pathogenic SIV Infections of Non-Natural Hosts (Asian NHPs)

Many common NHP models generate at least some macrophage infection in vivo (see Table 2), but levels of macrophage infection are greatly elevated in models where T-cells are rapidly depleted either by a highly pathogenic viral swarm [102,103,104,105] or by antibody depletion of CD4+ or CD8+ T-cells [106,107]. Table 2 summarizes the methods used to generate some of the SIVs most commonly used in NHP models of HIV and their tropism in vivo and in vitro.

### Macrophage-Tropic SIVs often Have the Ability to Infect Cells Lacking CD4

A common feature of both macrophage-tropic HIV-1 and SIV variants is that they have an increased ability to infect cells expressing low levels of CD4 [9,11,12,14,79,80,81,82], but they differ in a number of important ways. Most notably, macrophage-tropic SIVs are typically able to infect cells lacking CD4 [14,15,16,17,18], while patient-derived HIV-1 variants always require CD4 for efficient entry [89]. In addition, most macrophage-tropic SIVs are highly sensitive to neutralization [128]. This is illustrated by the observation that greater CD4-independence among SIV Envs is associated with increased neutralization sensitivity [18], suggesting that mutations that increase CD4-independence also expose epitopes. In contrast, patient-derived, macrophage-tropic HIV-1 is not significantly more sensitive to broadly neutralizing antibodies (bNABs) than T-cell-tropic HIV-1 [129].

The neutralization sensitivity of CD4-independent SIV Envs likely stems from continuous exposure of their coreceptor binding site [98]. This conformation allows CD4-independent Envs to bind their coreceptor without first binding CD4, thus facilitating entry into cells lacking CD4. Interestingly, macaques that are not CD4-depleted before infection and people chronically infected with HIV-1 have antibodies that recognize epitopes that are exposed on CD4-independent Envs [130,131], suggesting that CD4-independent Envs may be selected against in immunocompetent NHPs and in chronically-infected humans. There are, however, conditions where CD4-independent SIVs may have a fitness advantage. Specifically, when NHPs are infected with a macrophage-tropic SIV capable of efficient CD4-independent entry and are immunosuppressed, the resulting infections are often highly pathogenic, with large numbers of infected macrophages [106,107,111,122,123]. Under these conditions, immunosuppression may allow macrophage-tropic SIVs to evade immune recognition and colonize macrophage-rich tissues such as the CNS.

While many of the most commonly studied macrophage-tropic SIVs are capable of CD4-independent entry (Table 2), in vitro analyses of SIVsmE543-3, which was derived from a macaque with SIV-induced encephalitis, indicate that this variant is macrophage-tropic but retains the need for CD4 to efficiently enter host cells [15], thus raising the possibility that macrophage-tropic SIVs may either evolve under conditions that select for variants that are CD4-independent or under conditions that select for variants that are dependent on CD4 for efficient entry. This pattern could emerge if CD4-independent variants are only favored in highly pathogenic NHP infections with severe immunodeficiency. Alternatively, these two types of macrophage-tropic SIVs may represent different evolutionary pathways that evolved independently under the same selection pressures.

Consistent with the notion that macrophage-tropic Envs may be under intense immune pressure in immunocompetent hosts, SIV variants that are macrophage-tropic in vitro do not always generate extensive macrophage infection in vivo [115]. For example, a study examining animals infected with a variant that is macrophage-tropic in vitro (SIVmac316) found that the macrophage-tropic variant poorly infected macrophage in vivo. This discrepancy may partially reflect the fact that the macrophage-tropic variant was both highly sensitive to neutralization [128] and generated a less pathogenic infection with a much lower set-point viral load [115]. Thus, immune recognition of this neutralization-sensitive variant may have kept viral loads low and precluded viral colonization of macrophage-rich tissues.

While CD4-independent SIVs can replicate in vivo, they rarely infect cells lacking CD4. One exception is a pathogenic model in which pig-tailed macaques are coinfected with a highly immunosuppressive SIV viral swarm and a macrophage-tropic clone [15,16]. In this model, the virus frequently infects brain endothelial cells that lack CD4 [15,16]. The paucity of SIV-infected, CD4-negative cells in other model systems may indicate that CD4-independent entry is highly inefficient in vivo or that the phenotype is rapidly lost as the viral population adapts to the host environment.

## 5. Infection of Macrophages in Nonpathogenic SIV Infections of Natural Hosts (African NHPs)

SIV infection of natural hosts typically generates high levels of viral replication without progression to AIDS [6,132]. There are likely many factors that reduce SIV pathogenesis in natural hosts [132], with one hypothesis being that pathogenesis is reduced by blocking macrophage infection [20].

### 5.1. CD4-Dependence of SIV in Natural Hosts

SIVs are highly diverse and their cellular tropism has only been directly examined in a small number of natural hosts, but the available evidence suggests that CD4 usage is conserved in SIV infections of natural hosts [133,134]. For example, SIVs from sooty mangabeys (SIVsm and SIVsmmPBj) [133,134] and African green monkeys (SIVagm) [134] have been shown to require CD4 for entry. In contrast, coreceptor usage is less conserved, as illustrated by SIVrcm (from red-capped mangabeys), which utilizes the alternative coreceptor, CCR2 [135].

### 5.2. There are Substantial Blocks to SIV Infection of Macrophage in Their Natural Hosts

SIV-infected African NHPs typically do not experience highly pathogenic infections, and their macrophages are largely resistant to infection, thus raising the possibility that blocks to macrophage infection may help these natural hosts avoid the pathogenic effects of SIV. Mir et al. [20] assessed this hypothesis by comparing the ability of SIVm949 to infect MDM from natural hosts (sooty mangabeys) and non-natural hosts (rhesus macaques). SIVm949 replicated well in MDM from non-natural hosts, but poorly in MDM from natural hosts, despite the fact that it replicated equally well in PBMCs from the two hosts. This clearly illustrates a block in the ability of SIVm949 to infect MDM from the natural host. In order to further explore the mechanisms blocking macrophage (but not CD4+ T-cell) infection in the natural host, the authors quantified the expression of host restriction factors and found that multiple restriction factors (including tetherin and TRIM22) were expressed at higher levels in the natural host.

Are these blocks to macrophage infection in natural SIV hosts also observed in vivo? In a study of SIV-infected sooty mangabeys, depletion of CD4+ T-cells (but not macrophages) resulted in reduced plasma viral loads [136]. In contrast, depletion of CD4+ T-cells (but not macrophages) from non-natural hosts prior to infection resulted in extremely high viral loads, rapid progression to AIDS, and extensive infection of macrophages and microglia. These results indicate that SIV-infected macrophages can produce large amounts of virus in non-natural hosts but may contribute little to viremia in natural hosts [106,111]. Variation in macrophage tropism across natural hosts is unknown, however, analyses of genetic diversity in CD4 genes across African NHPs have identified extensive variation [137] that could potentially alter viral tropism, including macrophage tropism.

There is now both in vitro and in vivo data indicating that SIV infection of macrophages is blocked in natural hosts, but not in non-natural hosts. The ability of SIV to enter macrophages from non-natural hosts depends on the surface density of CD4 [14], but studies have not examined whether increasing CD4 expression facilitates entry into macrophages from natural hosts. Such information would be helpful to explore the contribution that pre- and post-entry mechanisms make to this block in macrophage infection.

## 6. Conclusions

SIVs are capable of generating a wide range of disease states, with highly variable levels of macrophage infection. Our understanding of how natural hosts are protected from the pathogenic effects of SIV infection remains unclear, but there is growing data suggesting that blocks to macrophage infection in natural hosts may contribute to this protection. However, the fact that some highly pathogenic SIV and HIV-1 infections do not generate high levels of macrophage infection suggests that extensive macrophage infection is not a requirement for the development of pathogenic infections. An alternative hypothesis is that extensive infection of macrophages is a symptom of highly pathogenic infections. Additional work is needed to untangle the mechanistic link between pathogenesis and infection of macrophages in primate lentiviruses.

## Figures and Tables

**Table 1:** Receptor and coreceptor expression on a subset of myeloid lineage cells and CD4+ T cells from humans and Asian NHPs.

Species	Potential Target Cells	CD4	CCR5	CXCR4	Citations
Humans	Monocyte-derived macrophages (MDM)	Positive (Low)	Positive (High)	Positive (Low)	[31,33]
	Tissue macrophages	Positive (Low)	Positive	Positive	[35,36,37]
	Monocytes	Positive (Low)	Positive (High)	Positive (High)	[31,33,34]
	DCs	Positive (Low)	Positive	Positive	[31,38,39]
	Follicular DCs	Negative	?	?	[40,41,42]
	Memory CD4+ T-cells	Positive (High)	Positive (High)	Positive (Low)	[31]
	Naïve CD4+ T-cells	Positive (High)	Negative	Positive (High)	[31]
Asian NHPs	MDM	Positive	Positive	Positive	[14]
	Tissue macrophages	Positive (Low to undetectable)	Positive	Positive	[43,44]
	DCs	Positive	Positive	?	[45]
	Memory CD4+ T-cells	Positive	Positive	Positive	[46]
	Naïve CD4+ T-cells	Positive	Negative	Positive	[46]
	monocytes	?	Positive	?	[47]

**Table 2:** Macrophage tropism of SIVs commonly used in NHP models of HIV infection.

Virus	Source	Infect Macaque Macrophage In Vitro	Efficiently Infect Macaque Macrophage In Vivo	Able to Infect Cells Lacking CD4
SIVmac251	Spleen cells were collected from an SIV-infected macaque who developed AIDS and then cultured in vitro. SIVmac251 is a viral swarm initially collected from this cell supernatant and amplified in macaque cells [108,109]	Yes, MDM [110]	Conflicting data, but clearly replicates when CD4+ or CD8+ T-cells are depleted [106,107,111,112]	Yes [15]
SIVmac239	Tissue from the infected animal used to derive SIVmac251 was in vivo passaged through additional macaques. Plasma from one of these animals was used to infect cells in vitro, and an infectious molecular clone was generated from the culture supernatant [108,109,113]	No, MDM [14,114]	Yes [115,116]	No [14,18]
SIVmac316	Alveolar macrophages (AM) were collected from a macaque infected with a SIVmac239-derived isolate. The AM were cultured in vitro and SIVmac316 was isolated from supernatant [117,118]	Yes, alveolar macrophages and MDM [14,117]	No [115]	Yes [14,18]
SIV/17E-Fr	SIVmac239 was passaged in macaques with brain homogenate from the first animal being used to infect the second. SIV/17E-Br was isolated from the brain of the second animal after it developed neurologic disease. The env, nef, and the 3’ LTR of SIV/17E-Br were cloned into SIVmac239 to generate SIV/17E-Fr [52,119,120]	Yes, MDM [52,120]	Yes [121], but infection is greatly enhanced in animals coinfected with SIV/DeltaB670 [122,123].	Yes [15,16,17,18]
SIVsmE543-3	Uncloned SIVsmF236 was used to inoculate a macaque who developed neurological disease. PBMCs were collected from this animal at the time of necropsy, and cell-associated virus was expanded in CEMx174 cells and then cloned to yield SIVsmE543-3 [124]	Yes, MDM [124,125,126]	Yes [124]	No [15]
SIVsm804E-CL757	SIVsmE543-3 was sequentially passaged in 4 rhesus macaques, and virus was isolated from the brain after passage [125,126]	Yes, MDM [125,126]	Yes [127]	?
